# Effect of Ceramic Thickness on the Bond Strength to Resin-Luting Agents before and after Thermal Cycling

**DOI:** 10.1590/0103-6440202405619

**Published:** 2024-03-22

**Authors:** Carolina Rodrigues de Souza, Ana Rosa Costa, Lincoln Pires Silva Borges, Analia Gabriella Borges Ferraz, Rafael Leonardo Xediek consani, Rafael Rocha Pacheco, Américo Bortolazzo Correr, Lourenço Correr-Sobrinho

**Affiliations:** 1 Department of Restorative Dentistry, Dental Materials Division, Piracicaba Dental School, UNICAMP, State University of Campinas, Piracicaba, SP, Brazil; 2 Department of Orthodontics, Graduate Program in Orthodontics - UNIARARAS, Universidade de Araras, SP, Brazil; 3 Department of Prosthodontics and Periodontology, Division of Complete Denture, Piracicaba Dental School, UNICAMP, State University of Campinas, Piracicaba, SP, Brazil; 4 Department of Restorative Sciences, The Dental College of Georgia at Augusta University, Augusta, GA, USA

**Keywords:** Ceramic, polymerization, resin-luting agents, bond strength, thermal cycling, dental materials

## Abstract

This study investigated microshear bond strength (µSBS) of two (2) dual-cured resin-luting agents (RelyX™ Ultimate and RelyX™ U200) when photoactivated through varying thicknesses of lithium disilicate, with or without thermal cycling. Discs of IPS e.max Press of 0.5, 1.5, and 2 mm in thickness were obtained. Elastomer molds (3.0 mm in thickness) with four cylinder-shaped orifices 1.0 mm in diameter, were placed onto the ceramic surfaces and filled with resin-luting agents. A Mylar strip, glass plate, and load of 250 grams were placed over the filled mold. The load was removed and the resin-luting agents were photoactivated through the ceramics using a single-peak LED (Radii Plus.) All samples were stored in distilled water at 37^o^C for 24 h. Half of the samples were subjected to thermal cycling (3,000 cycles; 5ºC and 55ºC). All samples were then submitted to µSBS test using a universal testing machine (Instron 4411) at a crosshead speed of 0.5 mm/min. Data were submitted to three-way ANOVA and Tukey post-hoc test (α=0.05). The mean µSBS at 24 h was significantly higher than after thermal cycling (p<0.05). No statistical difference was found between resin-luting agents (p > 0.05). The mean µSBS for groups photoactivated through 0.5 mm ceramic were significantly higher than 1.5 mm and 2.0 mm (p < 0.05). In conclusion, increased ceramic thicknesses reduced the bond strength of tested resin-luting agents to lithium disilicate. No differences were found between resin-luting agents. Thermal cycling reduced the bond strength of both resin-luting agents.

## Resumo

Este estudo investigou a resistência de união ao microcisalhamento (RUµC) de dois (2) agentes de cimentação de resina dual (RelyX™ Ultimate e RelyX™ U200) quando fotoativados através de diferentes espessuras de dissilicato de lítio, com ou sem ciclagem térmica. Discos do IPS e.max Press de 0,5, 1,5 e 2 mm de espessura foram obtidos. Moldes de elastômero (3,0 mm de espessura) com quatro orifícios cilíndricos de 1,0 mm de diâmetro foram colocados sobre as superfícies cerâmicas e preenchidos com agentes de cimentação de resina. Uma tira Mylar, placa de vidro e carga de 250 gramas foram colocadas sobre o molde preenchido. A carga foi removida e os agentes de cimentação resinosos foram fotoativados através da cerâmica usando um LED de pico-único (Radii Plus). Todas as amostras foram armazenadas em água deionizada a 37^o^C por 24 h. Metade das amostras foi submetida a ciclagem térmica (3.000 ciclos; 5ºC e 55ºC). Todas as amostras foram então submetidas ao teste de RUµC usando uma máquina de teste universal (Instron 4411) com velocidade de 0,5 mm/min. Os dados foram submetidos à Análise de Variância três fatores e ao teste post-hoc de Tukey (α = 0,05). A média de RUµC em 24 h foi significativamente maior do que após a ciclagem térmica (p < 0,05). Não houve diferença estatística entre os cimentos resinosos (p > 0,05). As médias de RUµC para grupos fotoativados através de cerâmica de 0,5 mm foram significativamente maiores do que 1,5 mm e 2,0 mm (p < 0,05). Em conclusão, o aumento da espessura da cerâmica reduziu a resistência de união dos agentes de cimentação resinosos ao dissilicato de lítio. Não foram encontradas diferenças entre os agentes de cimentação resinosos. A ciclagem térmica reduziu a resistência de união de ambos os agentes de cimentação resinosos.

## Introduction

Dental ceramics have been extensively employed in dental procedures to restore dental tissues that have been lost due to caries disease, fracture, genetic malformation, and/or aesthetic reasons. These restorative materials must have properties that mimic the natural substrates of the tooth, such as mechanical strength under varying stresses, wear resistance, biocompatibility, and color stability ^1^. Indirect or direct restorations typically consist of layers of different materials, resulting in a restoration system with multiple interfaces ^2^. The minimally invasive restorative approach proposes procedures that protect pulpal tissues and preserve as many healthy tooth structures as possible ^3^.

The clinical success and longevity of minimally invasive procedures using dental ceramics tend to rely on the quality of the adhesive interface, which is directly related to the material properties and clinical cementation technique ^4^. Glass ceramics can adhere to a dental substrate using adhesive techniques that involve the use of adhesive systems and a resin-luting agent ^5^. Various surface treatments for glass ceramics have been reported to increase their adhesion to polymeric materials. The application of hydrofluoric acid and silane is the most recommended method ^6^. Hydrofluoric acid improves the wettability and micromechanical bond strength between ceramic and resin-luting agents ^6^ ^,^ ^7^ by increasing the surface roughness and, thus, the surface area and surface energy of the ceramic. The silane also facilitates the chemical bond between resin-luting agent and glass ceramics, as the silanol group binds to the SiO groups present in these glass ceramics ^6^.

By distributing stresses along the bonded interface using dental adhesive systems ^8^, adhesive cementation allows the clinician to carry out a restorative procedure that behaves biomechanically as a single structure with the tooth. In addition, the resin-luting agent can penetrate microcracks in the restorative material, thereby reducing crack propagation ^9^ and secondary stresses ^10^. Resin-luting agents are essentially polymeric materials with lower solubility than other dental cement. These materials' desirable properties include ease of manipulation, handling, biocompatibility, and low technique sensitivity ^11^ ^,^ ^12^. According to their chemical composition and clinical application, resin-luting agents can be categorized as conventional, self-etching, or self-adhesive. Conventional resin-luting agents are compatible with total-etch adhesive systems (use of 30-40% phosphoric acid on dentin, moist technique), whereas self-etch resin-luting agents are compatible with self-etch adhesive systems (dentin is not treated with phosphoric acid, dry technique). Self-adhesive resin-luting agents do not require association with an adhesive system, reducing the number of steps and technique sensitivity ^13^ ^,^ ^14^.

Resin-luting agents can also be categorized based on their curing method: [LC] light-cured, [SC] self-cured (chemically activated), and [DC] dual-cured (which employs both methods). Light-cured resin-luting agents have superior color stability compared to self- or dual-cured materials due to the absence of a tertiary amine as an initiator and improved control of working time ^15^. However, the limited blue-light penetration through dental substrates restricts the use of these resin-luting agents in certain procedures, which are influenced by the substrate's composition and thickness ^16^ ^,^ ^17^. The amount of energy that reaches the photoinitiators of the resin-luting agent is reduced, which can result in a reduced degree of conversion as well as modifications to the properties of these materials, thereby affecting longevity ^18^. 

Clinically, these restorative procedures are subject to multiple challenges, such as fatigue, thermal cycling, and water solubility/sorption that can alter the physical and mechanical properties of the materials over time ^8^. Some research methods, such as aging and storage, have been suggested to speed up the breakdown of these materials and the adhesive interface ^4^ ^,^ ^5^. Thermal cycling methods simulate the degradation mechanisms that can result in a decrease in bond strength due to stresses at the adhesive interface ^19^ ^,^ ^20^. This effect occurs as a result of differences in the coefficient of thermal expansion of materials affected by changes in temperature ^21^. However, little is known about the effect of aging on the bond strength between self-adhesive resin cement and ceramics of different thicknesses.

In this context and considering that many questions remain regarding bonding to ceramic with different thicknesses and self-adhesive resin cement after aging, the current study aimed to investigate the effect of light-curing two distinct resin-luting agents (self-etch and self-adhesive) through varying thicknesses of a glass ceramic (lithium disilicate) on the microshear bond strength (µSBS), with or without thermal cycling. The tested hypotheses were: [1] ceramic thickness will influence µSBS; [2] distinct resin-luting agents will influence µSBS; and [3] thermal cycling will influence µSBS.

## Materials and Methods

### Specimen preparation

A self-etch dual-cured resin-luting agent (RelyX™ Ultimate, 3M ESPE, St. Paul, MN, EUA) and a self-adhesive dual-cured resin-luting agent (RelyX™ U200, 3M ESPE) were evaluated in this study. A total of ninety-six ^9^, ^6^ lithium disilicate ceramic specimens with a diameter of 12.0 mm and a thickness of 2.2 mm were manufactured (IPS e. max Press Impulse, Ivoclar Vivadent, Schaan, Liechtenstein). To fabricate the discs, acrylic resin patterns were fabricated (Reliance Dental MFG Company, Illinois, USA) and invested in a phosphate-based investment (IPS PressVest Speed, Ivoclar Vivadent), followed by pattern elimination on a specific oven (Vulcan A-550, Degussa-Ney, Yucaipa, CA, USA) at 950ºC for 60 minutes. The mold was filled with ceramic ingots that were injected using specialized equipment (EP600, Ivoclar Vivadent) at 915°C. Ceramic specimens were extracted from investment and finished/polished using SiC sandpaper (Norton SA, Sao Paulo, SP, Brazil) with progressive grit (#400, #600, #1200) in a water-cooled polisher (APL4; Arotec, Cotia, SP, Brazil). The thicknesses of the specimens were measured using a digital caliper (Mitutoyo, Tokyo, Japan) and ultrasonically cleaned (MaxiClean 750, Unique, Indaiatuba, SP, Brazil) with deionized water for five ^5^ minutes. Specimens were separated into two ^2^ groups (n = 48) based on the resin-luting agent and further subdivided into three ^3^ groups (n = 16) based on ceramic thickness. The surfaces of the ceramic discs were etched with 10% hydrofluoric acid (Dentsply Caulk, Milford, DE, USA) for 20 seconds, followed by 30 seconds of water rinsing and 30 seconds of air-drying. The surfaces were coated with two layers of silane (RelyX™ Ceramic Primer, 3M ESPE) and allowed to evaporate at room temperature for 60 seconds, followed by 30 seconds of air-drying with a syringe.

### Thermal cycling and microshear bond strength (µSBS)

To create the resin-luting agent specimens for µSBS, polyvinylsiloxane (PVS) molds (3.0 mm in thickness) with four ^4^ cylindrical perforations (1.0 mm in diameter each) were fabricated. On the silane-treated ceramic surface (intaglio), a thin layer of bond (Scotchbond™ MultiPurpose, 3M ESPE, Seefeld, Germany) was applied to the entire surface, followed by light-curing for 10 seconds using an LED light curing unit (LCU) (Radii Plus, SDI Limited, Bayswater, Victoria, Australia) with an emitted irradiance of 1,100 mW/cm^2^ (Radiometer, model 100, Demetron Research Corporation, Danbury, CT). The molds (Express STD, 3M ESPE) were placed on the intaglio surfaces, and the orifices were filled with a resin-luting agent according to the experimental group and manufacturer's instructions. A mylar strip and a glass plate were placed on top of the filled mold, then a 250g load was applied for two ^2^ minutes ^11^. After removing the load, the resin-luting agents were photoactivated through the ceramic using the same LCU for 20 seconds with the light tip in direct contact with the ceramic's outer surface. Molds were removed using a scalpel, and all specimens were stored for 24 hours at 37ºC in distilled water. Half of the samples were then thermally cycled (3,000 cycles) in a thermal cycler (MSCT 3 - Marnucci ME, São Carlos, SP, Brazil) with water between 5ºC and 55ºC (dwell time of 30 seconds) and transfer time of 6 seconds between baths. Using cyanoacrylate glue, the outer ceramic surface was attached to a device. The apparatus was placed on a universal testing machine (Instron 4411, Instron Co., USA) and a steel wire (0.2 mm in diameter) was looped around each cylinder and aligned with the bonding interface. The µSBS test was performed at a crosshead speed of 0.5 mm/min until failure. There were no pre-test failures.

### Failure mode

The de-bonded specimens were examined by optical microscopy (Olympus Corp, Tokyo, Japan) at 40X magnification, and the failure modes were classified as follows: adhesive between resin-luting agent and ceramic (mode 1), cohesive within ceramic (mode 2), cohesive within resin-luting agent (mode 3), and mixed, involving resin-luting agent and ceramic (mode 4). 

### Statistical analysis

The average of each specimen was determined using four ^4^ resin cylinders. Thus, the average µSBS values for each group represented the average of eight experimental units. Before a three-way ANOVA analysis (cement x thickness x aging condition), data were examined for normality (Shapiro-Wilk) and variance equality (Levene). The Tukey post-hoc test (α = 0.05) was used to perform multiple comparisons using SPSS. The failure mode classification results were analyzed using Fisher's Exact Test (α = 0.05).

## Results

### Microshear bond strength (µSBS)

Table 1 reveals significant differences in µSBS based on aging condition (p < 0.0001) and ceramic thickness (p < 0.0001). There was no difference between the resin-luting agents (p = 0.608). The interaction between resin-luting agents and ceramic thickness was statistically significant (p < 0.011). The interaction between resin-luting agents and aging conditions was not significant (p = 0.448). The interaction between the aging condition and ceramic thickness was not significant (p = 0.813). The interaction among the three factors was not significant (p = 0.526). After 24 hours, the mean values of µSBS were significantly higher than after thermal cycling (p > 0.05). There were no statistically significant differences (p > 0.05) between RelyX™ Ultimate and RelyX™ U200 resin-luting agents. The µSBS through a ceramic thickness of 0.5 mm was significantly greater than 1.5 mm and 2.0 mm (p < 0.05). The lowest µSBS was identified at 2.0 mm. Compared to the ceramic thickness of 0.5 mm, the thicknesses of 1.5 mm and 2.0 nm resulted in a 21.2% and 27.6% reduction in µSBS, respectively. In comparison to 24 hours, thermal cycling reduced µSBS by 9%. 

### Failure mode

**Figure 1:**
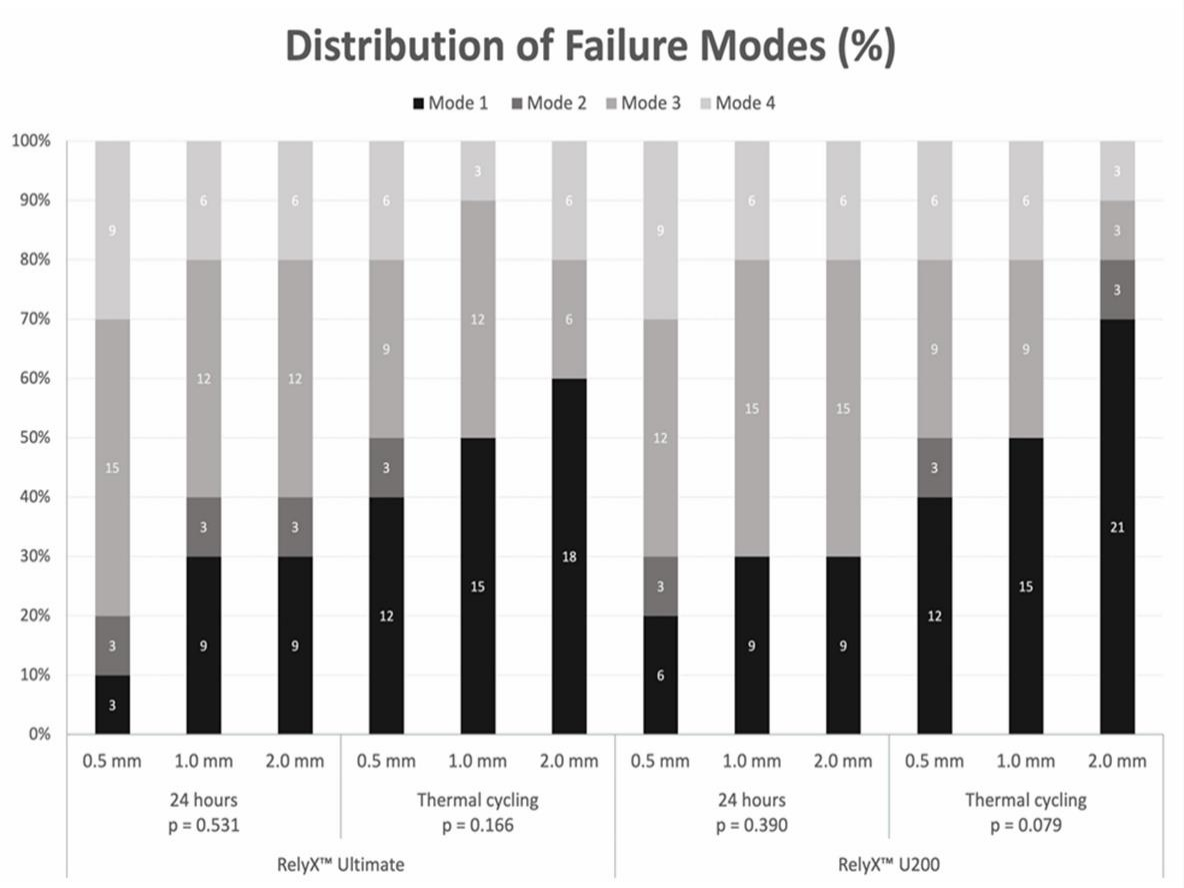
Failure mode analysis of the debonded specimens. Failure modes: adhesive between cement and ceramic (mode 1); cohesive within ceramic (mode 2); cohesive within resin cement (mode 3); and mixed, involving resin cement and ceramic (mode 4).

Figure 1 displays the failure mode results. Despite a greater tendency for adhesive (mode 1) failures after thermal cycling as compared to 24 hours, Fisher's Exact Test of the failure modes within each condition revealed no significant association between the failure modes and ceramic thickness for each resin-luting agent (p > 0.05). It is possible to observe a higher percentage of adhesive failures (mode 1) and cohesive within resin-luting agent (mode 3).

**Table 1:** Means of µSBS ± Standard Deviation (MPa) for all groups. Parenthetical values under Tukey indicate the overall mean bond strength for the indicated ceramic thickness.

Treatment^a^	Ceramic	Resin Cement^b^		Tukey^a^
	Thickness	RelyX U200	RelyX Ultimate	
24 hours (28.5)^a^	0.5 mm	33.6 ± 1.1	34.5 ± 1.7	
	1.5 mm	27.6 ± 0.6	26.1 ± 1.6	0.5 mm (32.6)^a^
	2.0 mm	25.1 ± 1.6	24.2 ± 3.0	1.5 mm (25.6)^b^
Thermal cycling (25.9)^b^	0.5 mm	29.6 ± 0.4	32.5 ± 1.4	2.0 mm (23.6)^c^
	1.5 mm	24.9 ± 1.8	23.9 ± 3.0	
	2.0 mm	23.1 ± 2.9	21.6 ± 2.4	
Tukey		27.4 A	27.1 A	

a Means followed by different lowercase letters indicate significant differences for treatment and for ceramic thickness (p<0.05).

b Same capital letters indicate no significant differences between resin cement (p>0.05).

## Discussion

The first hypothesis of this study, that ceramic thickness will influence µSBS, was accepted. The results demonstrated that the µSBS in groups where the resin-luting agents were light-activated through a 0.5mm ceramic were significantly greater than the values observed for 1.5mm and 2.0mm thicknesses, with a 21.2% and 27.0% reduction in bond strength, respectively. This result is consistent with previous studies showing a significant decrease in the amount of incident irradiance reaching the adhesive interface as a result of a decrease in light transmission with increasing ceramic thickness ^17^ ^,^ ^22^. Studies indicate that the increased thickness of the material has a direct impact on the amount of dispersion, scattering, reflection, and absorption of emitted light ^23^ ^,^ ^24^. As the thickness of a ceramic increase, scattering and absorption may reduce light transmission exponentially ^23^. However, another study showed that indirect activation through ceramic has no significant effect on the degree of conversion of dual-cured resin-luting ^25^.

As the polymerization process is dependent on the emitted irradiance and exposure time, low radiant energy may influence the degree of conversion of polymeric materials by decreasing double-bond conversion. These materials contain photoinitiators that are sensitive to specific wavelengths and are activated by specific radiant energy values ^26^ ^,^ ^27^. Studies indicate that polymerization is properly induced and propagated when enough free radicals are released. This effect is directly proportional to the radiant energy at specific wavelengths (camphorquinone’s peak of absorption is 468nm) ^28^ ^,^ ^29^. Lower degrees of conversion can affect mechanical properties, including an increase in the solubility of the resin-luting agent ^16^ ^,^ ^30^, which can result in ceramic surface debonding over time ^31^. Light curing units with higher irradiance emissions at the correct wavelengths likely promote the formation of densely cross-linked networks ^32^ by increasing the number of polymer-growth centers. Consequently, polymers with greater levels of cross-linking may result in higher bond strengths.

The second hypothesis was rejected as there was a distinct resin-luting agent that would influence µSBS, regardless of ceramic thickness and aging. This study demonstrated that, when light-activated under identical conditions, the two dual-cure resin-luting agents exhibited similar behavior. This is most likely due to the similar composition of the resin-luting agents, with minor differences in the components such as filler, monomers, and catalyst paste (same manufacturer). Previous research has demonstrated that the composition of cement, such as the monomer type and inorganic particle content, affects the polymerization reaction ^33^. Another study has identified a significant difference between a self-etch dual-cured resin-luting agent and a self-adhesive dual-cured resin-luting agent when light-activated through ceramic interposition under identical conditions ^34^. Another study showed that conventional resin cement does not differ significantly between groups, with or without disc interposition at 20 minutes after mixing ^35^. This may be due to the quantity of photoinitiators and their sensitivity during light curing through the disc ^36^. In contrast, the self-etch dual-cured resin-luting agent differs significantly between groups, with or without disc interposition at 20 minutes after mixing ^35^. Resin-luting agents containing acidic monomers may also have a negative effect on the degree of conversion of dual-cured materials, especially in the self-curing mode ^37^. The self-adhesive resin cement presents a lower extent of C=C conversion, as well as a slower rate of polymerization, than the conventional resin cement, irrespective of the curing mode ^37^. In addition, the self-adhesive resin cement needed more time to achieve its maximum conversion than did the conventional resin cement, irrespective of the curing mode ^37^.

The third tested hypothesis was accepted because the µSBS results for 24 hours were significantly greater than those after thermal cycling, consistent with previous studies ^21^. Differences in the coefficients of thermal expansion and conductivity of different materials, particularly at the interfaces ^19^, can amplify the effect of stresses induced by temperature variations. In addition, a material with a different modulus of elasticity at the adhesive interface may concentrate stress and increase degradation ^38^ ^,^ ^39^. These factors, in conjunction with the effect of continuous water sorption during mechanical testing, may have contributed to the µSBS reduction. After thermal cycling, the bond strength decreased by 9% compared to the 24-hour storage group. Analysis of failure modes revealed a greater number of cohesive failures (mode 3) in resin-luting agents at 24 hours for all ceramic thicknesses and resin-luting agents. A greater number of adhesive failures (mode 1) were observed after thermal cycling, indicating that the thermal cycling challenges had a negative impact on the adhesive system.

The observed results for µSBS and analysis of failure mode indicate that the ceramic thicknesses and thermal cycling challenges have significant effects on the quality and durability of adhesive interfaces. Yet, the type of dual-cured resin-luting agent utilized did not affect the results. Thus, future research should be conducted to evaluate other possible factors that could affect the quality and durability of adhesive interfaces, such as mechanical cycling (fatigue), resin-luting agent viscosity, and ceramic surface treatments.

In summary, the bond strength values of various resin-luting agents that were light-activated through ceramic interpositions decreased as their thickness increased. The different dual-cured resin-luting agents had no impact on the bond strength. At all ceramic thicknesses, thermal cycling reduced the bond strength of both resin-luting agents.
